# #Nomask on Instagram: Exploring Visual Representations of the Antisocial Norm on Social Media

**DOI:** 10.3390/ijerph19116857

**Published:** 2022-06-03

**Authors:** Yunhwan Kim

**Affiliations:** College of General Education, Kookmin University, Seoul 02707, Korea; yunhwankim2@kookmin.ac.kr

**Keywords:** #NoMask, Instagram, social representation theory, hashtag, k-means clustering

## Abstract

Social media (SM) functions such as hashtags and photo uploading can enrich and expedite user interactions, but can also facilitate the online spread of antisocial norms. Mask aversion is one such antisocial norm shared on SM in the current COVID-19 pandemic circumstances. This study utilized the social representation theory (SRT) to explore how mask aversion is visually represented in the Instagram photos tagged with #NoMask. It examined the overall content of the photos, the characteristics of the faces portrayed in the photos, and the presented words in the photos. Additionally, the study grouped the photos through k-means clustering and compared the resulting clusters in terms of content, characteristics of the faces, presented words, pixel-level characteristics, and the public’s responses to the photos. The results indicate that people, especially human faces, were visually represented the most in the Instagram photos tagged with #NoMask. Two clusters were generated by k-means clustering—Text-centered and people-centered. The visual representations of the two clusters differed in terms of content characteristics and pixel-level attributes. The texts presented in the photos manifested a unique way of delivering key messages. The photos of the people-centered cluster received more positive comments than the text-centered one; however, the two clusters were not significantly different in eliciting engagement. This study can contribute to expanding the scope of SRT to visual representations and hashtag movements.

## 1. Introduction

Users of social media (SM) can utilize the functions of platforms to interact with others in easy and diverse ways [1,2,3]. For example, they can upload posts in multimedia formats that allow them to share their ideas and feelings with other users via photos and videos. Photo-centric SM such as Instagram bolstered this tendency, and photo-sharing has now become a major cultural trend [4,5]. Users can also use the hashtag function that enables them to discover posts related to their interests and conveniently engage in public discussions. Twitter pioneered the use of the hashtag feature to help users find posts of interest, but users widened the scope of the hashtag to express their stances on varied social issues [6].

Such easy and diverse interactions through SM functions do not always lead to socially desirable outcomes [7]. Various antisocial norms [8] can easily be articulated, shared, reinforced, and practiced using SM functions [9]. Particularly, photos can facilitate the wide and fast dissemination of antisocial norms much more than text because visual media can portray stimulating content that exerts a stronger impact than textual media. Researchers have investigated antisocial norms and their visual depictions on SM, such as through images and multimedia content concerning self-harm [10], antivaccination [11], alcohol use [12], and cigarette smoking [13].

Mask aversion can be cited as an antisocial norm that has emerged during the current COVID-19 pandemic. The refusal to observe preventive rules, such as social distancing and mask-wearing, can directly and indirectly risk public health [9]. Wearing face masks is evidentially effective in reducing the risk of COVID-19 infections [14], but mask aversion is still projected as an antisocial norm online. The extant literature has noted varied aspects of mask aversion, including topics of discussion [15], sentiments [16], linguistic characteristics [17], user networks [18], and opinion polarization [19]. However, scholars have not yet examined how the mask aversion norms are visually represented in photos uploaded on SM.

In this regard, the present research aims to explore the visual representations of mask aversion as an exemplar of antisocial norms disseminated on SM. Instagram photos with #NoMask hashtag are downloaded and analyzed to achieve this objective. The social representation theory (SRT) [20] is employed for the analysis. This concept is introduced below, focusing on the hashtag and visual representations. Apart from the overall exploration, this study also aims to identify the major components of visual representations and reveal how they differ. The clustering method is adopted as the analytical strategy to accomplish this task, and this technique is also briefly overviewed below.

### 1.1. Social Representation Theory, Hashtags, and Visual Representations

SRT is concerned with the common knowledge of a particular object in a society and the interactions through which the knowledge is created among people [21]. It deals with a social representation (SR), which is an ensemble of thoughts and feelings manifested through various verbal and nonverbal articulations and behaviors [22]. This SR serves the following two major functions in a society [23]: it facilitates the interpretation of social objects, and it enables individuals to communicate about the objects and to signify their relationships with them. An SR is generated by many individuals; therefore, it can include fragmented thoughts and contradictory ideas rather than logical and coherent notions [24].

SRT can offer a theoretical lens for the analysis of hashtagged posts on SM, because hashtags can represent an SM function through which SRs are created and disseminated online. The word to which the # (hash) sign is attached to create a hashtag usually concerns an object of interest for SM users. This hashtag is spread when many users attach it to their posts expressing diverse ideas and opinions on the object of interest. The hashtag can also be used as a search query to discover posts on a particular topic. Thus, posts with a hashtag may be deemed a channel through which SM users interact with others and create SRs of an object, and the analysis of such posts would denote a promising approach toward attaining an understanding of SRs on SM.

SRs are shaped in visual as well as verbal forms. Visual materials were pivotal to the original formulation of SRT [25], and visual data analysis exhibits an increase in recent SR research [26,27,28]. Visual materials communicate their meanings in ways that differ from verbal messages. The link between a verbal symbol and its referent is not based on similarities in shape; however, a visual symbol is directly connected to its referent through an analogous appearance [27]. Thus, SRs mediated in visual forms should be investigated with a focus on their visual characteristics. Such evaluations would manifest aspects of visual SRs that are distinguished from verbally mediated ones. Visual representations of varied objects were examined in the literature in such a context. For example, groups of people in relation to the COVID-19 pandemic [29], sustainable energy [30], identity in a multicultural community [31], far-right groups on Facebook [32], and happiness [33] were investigated in terms of their visual representations based on SRT.

SRT suggests two key processes through which an SR is developed: anchoring and objectification [20]. Anchoring involves the naming and classification of novel ideas or objects to connect them to existing ones and to make the unknown familiar. Objectification renders abstract ideas tangible by presenting images, exemplars, and metaphors from everyday life [24]. The two processes discharge vital roles in the analysis of the SR of an object. Some studies separately investigated anchoring and objectification as independent processes [33]. However, the two processes are inherently linked, especially in the examination of photo data [26,29]. The photos of an object can reveal both how the object may be understood vis-à-vis existing knowledge (anchoring) and how it can be visualized to make it tangible (objectification).

This study adopted the following analytical strategies to investigate the visual representations in #NoMask Instagram photos. First, it examined the content of the photos. Content is a central concern of SRs and varying methodologies have been applied in SR research to grasp the substance of given materials [34]. Next, it investigated the characteristics of human faces in the photos. Faces denote the major objects in photos from the early history of Instagram [35]. They also serve as the major outlets of emotions, that shape the SRs of an object [24]. Finally, it evaluated the words presented in the photos. Previous studies have analyzed SRs in photos using caption texts accompanying the photos [33]. However, texts represent another major object in Instagram photos [35] and they may function differently from caption texts [11]. Thus, texts presented in photos can be deemed the major elements of visual representations. The following research questions are addressed based on the stated considerations:

RQ1. What is visually represented in the Instagram photos with #NoMask hashtag?

(a) What is the content of the photos?
(b) What are the characteristics of the faces in the photos?
(c) What words are presented in the photos?

### 1.2. Clustering as an Analytical Strategy for the Examination of Visual Representations

SRT is known for its flexibility in research methods and has been coupled with various techniques [36]. The same applies to investigations of visual SRs. Research methods such as discourse analysis [32], thematic analysis [33], and rhetoric approach [37] have been primarily applied in the extant literature using SRT to analyze visual data.

This study employs a clustering method to divide the dataset into subgroups and examines visual SRs at the subgroup level as well as at the level of the entire dataset, because an SR may comprise multiple distinct or even contradictory ideas and portrayals. Thus, identifying and comparing subgroups would better manifest the composition of the SR.

Clustering is an unsupervised learning technique which splits a dataset into subgroups by assigning similar units into the same group [38]. Subgroups can display latent structures or patterns in the dataset; thus, clustering has been used in the literature to analyze SM data [39]. In particular, clustering methods were used to analyze SM photos in order to investigate the events represented in geo-tagged tweets [40], the image of the Tri-City region in Poland [41], and vaping in Instagram photos [13].

The selection of features used to cluster a dataset is a crucial issue. Notably, this study uses different features to cluster photos and to compare the resulting clusters. Previous studies have reported that features extracted from deep neural networks (DNNs) performed better to cluster documents than manually extracted features [42]. Thus, this study uses DNN-based features for photo clustering. However, DNN-based features evince the weakness of their meanings being difficult to understand, and differences among clusters cannot be clearly revealed through such features. Thus, easier-to-understand photo features are extracted manually and are used to compare subgroups generated via the clustering method. The clustering procedure and extracted features are detailed in the methods section.

As an analytical strategy, the same criteria are employed to examine the entire dataset and compare clusters: content, facial characteristics, and words presented in photos. Additionally, clusters are compared using pixel-level features extracted from the photos. Visual representations encompass distinctive aspects that are difficult to articulate verbally [27]. Visual materials convey and create meaning not only through content-level characteristics but also through low-level characteristics, such as color and light, which are manifested as pixel-level features [29]. Also, clusters are compared with respect to the public’s responses. Previous studies have reported that the public responded differently based on the characteristics of photos on SM [43,44]. Thus, this study examines how the clusters, which may reveal the different aspects of visual SRs, would induce distinct public reactions. The following research questions are probed in the above-mentioned context:

RQ2. How do the clusters of Instagram photos with a #NoMask hashtag differ in terms of their content, faces in the photos, presented texts, pixel-level characteristics, and the public’s responses?

## 2. Methods

The overall procedure of this research can be summarized as follows:

- gather data
- investigate visual representations in the whole dataset: content, facial characteristics, and texts in photos
- cluster photos into subgroups
- compare subgroups in terms of visual representations: content, facial characteristics, texts in photos, pixel-level characteristics, and the public’s response

### 2.1. Research Sample

Public Instagram posts with a #NoMask hashtag were searched and downloaded using Instagram-scraper (https://github.com/arc298/instagram-scraper; accessed on 22 July 2020). Photos and accompanying information including likes and comments were downloaded. Ultimately, 32,104 photos, 125,875 comments, and 3,045,087 likes in total were aggregated for analysis.

### 2.2. The Content of Photos

The content of the collected photos was analyzed in two ways: (1) the frequency of the photos by content category and (2) the mean confidence scores of content tags. These computations were performed using Computer Vision API (application programming interface) in Microsoft Azure Cognitive Services (CV API; https://azure.microsoft.com/services/cognitive-services/computer-vision/; accessed on 24 March 2021).

To ascertain (1), each photo was categorized by the pretrained artificial intelligence (AI) based on its content into one of 15 predetermined classes: *abstract, animal, building, dark, drink, food, indoor, others, outdoor, people, plant, object, sky, text*, and *transportation*. The overall content of the photo corpus was then expressed using the frequency of the classes.

To determine (2), content tags and accompanying confidence scores were attached to each photo by the pretrained AI. A single class was assigned to a photo in (1), but multiple tags describing its content could be attached to a photo, and each tag was accorded a confidence score demonstrating the degree of correspondence between the tag and the photo content. The higher the confidence score of a tag, the more the content of a given photo pertained to the tag. For example, the content of a photo could be described as a set of tags with confidence scores: text (0.9999137), grass (0.999893069), outdoor (0.9880197), bicycle (0.9697462), sign (0.8423048), and land vehicle (0.6875147). The confidence scores of each tag were respectively averaged for all photos to evince the overall content of the photo corpus.

### 2.3. The Characteristics of Faces in Photos

Human faces appearing in a given photo were detected and their features were extracted using Face API in Microsoft Azure Cognitive Services (https://azure.microsoft.com/services/cognitive-services/face/; accessed on 24 March 2021). The pretrained AI estimated the age, gender, and expressed emotions from each detected face. The following features were used for analysis. The *number of faces* in a photo was ascertained, the *closeup* denoting the proportion of the largest face in the photo was determined, and the *face ratio* was calculated as the proportion of the sum of all faces in a photo. The average *age* of all faces was computed, and *gender* was designated via the number of female faces in the photo. The emotion detection in Face API is based on the basic emotion theory [45,46]: it suggests that human beings have a certain number of emotions which become the basic units of various emotional actions. Face API has been reported to be as accurate as human in detecting emotions from facial expressions [47] and used in the literature that analyzed SM data [48]. The emotions were expressed by a set of real numbers between zero and one, each of which corresponded to one of eight emotion classes: *anger*, *contempt*, *disgust*, *fear*, *happiness*, *sadness*, *surprise*, and *neutral*. The sum of all numbers was assigned to a given face. Each emotion class was then respectively averaged for all the faces in a photo.

### 2.4. Texts Presented in Photos

The English words appearing in a given photo were detected using the Optical Character Recognition function of CV API. The detected words were transformed into lowercase, and punctuation marks and Uniform Resource Locators were removed. Stop words (a, about, am, an, and, are, as, at, be, by, but, de, e, for, from, in, is, it, la, o, of, on, or, so, that, the, this, to, up, was, •, 0, 1, and 2) that occurred frequently but conveyed lesser meaning were removed. The frequency of the remaining words was then examined.

### 2.5. Clustering

**Figure 1:**
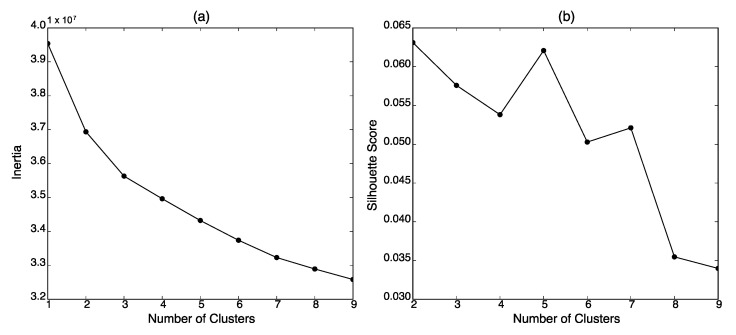
Determining the optimal number of clusters via k-means clustering using (**a**) the elbow method and (**b**) the silhouette score method.

The photos in the research sample were clustered through k-means algorithm [49]. First, each photo was transformed into a vector using the img2vec-keras library (https://github.com/jaredwinick/img2vec-keras; accessed on 29 September 2021). A photo was infused into the ResNet50 model [50] which was trained on the ImageNet [51] dataset, and the penultimate layer of the model was used as the vector of the photo with 2048 dimensions. Next, the optimal number of clusters was determined using the elbow method and the silhouette score method [52]. Figure 1 indicates that the elbow (a) and the highest silhouette score (b) were observed at the optimal number two. The photos were grouped into two clusters on the basis of this result: 8084 photos were placed in cluster 1 and 24,020 photos were allocated to cluster 2.

### 2.6. Pixel-Level Features

Colors can be expressed in digital photos by a color space model such as RGB (red, green, and blue) and HSV (hue, saturation, and value), and the following features were extracted from pixels in relevant formats using custom Python script and OpenCV library (https://opencv.org; accessed on 2 April 2021). These features were used to compare the pixel-level characteristics of photos between clusters.

The means and variances of the red, green, and blue in RGB and the saturation and value (i.e., lightness) in HSV were respectively calculated, and the following features were extracted: *red mean, red variance, green mean, green variance, blue mean, blue variance, saturation mean, saturation variance, value mean*, and *value variance*. Hue is a nominal feature unlike saturation and value, so its histogram was used for feature extraction instead of mean and variance. The hue histogram was smoothed by kernel density estimation and was measured by the number of its peaks (*hue peaks*) to apprehend the monotonousness or mussiness of a given photo [53].

Additionally, features regarding the visual attractiveness [54] of a given photo were extracted. First, *brightness* designates the brilliance of a photo and was measured by the average of luminance (Y values in the YUV color space) value of the pixels of the photo. Next, *colorfulness* designates the use of multiple hues in a photo and was calculated using Hasler and Süsstrunk’s [55] formula. *Naturalness* represents the extent of correspondence a photo displays to the human perception of reality, and this attribute was measured using Huang et al.’s [56] formula. *Contrast* signifies the relation of local luminance variations to the surrounding luminance, and this element was calculated through the standard deviation of luminance in the pixels of a given photo divided by the number of pixels of the photo. *RGB contrast* denotes the extension of contrast into the three-dimensional RGB color space. *Sharpness* designates the clarity and level of detail visible in a photo, and this quality was measured as a function of Laplacian of each pixel’s luminance, normalized by the local average luminance in the surroundings of every pixel.

### 2.7. The Public’s Responses

Public reactions to each photo were evaluated using two aspects: *engagement* and *comment sentiment*. Engagement was gauged by the sum of the number of likes and comments. The sentiment of each comment was examined using the Flair module (https://github.com/flairNLP/flair; accessed on 5 September 2021) of Python language. The pretrained model estimated the sentiment as a score between −1 (most negative) and 1 (most positive). All sentiment scores allotted to a photo’s comments were averaged and used for analysis.

## 3. Results

### 3.1. Visual Representations in the #NoMask Instagram Photos (RQ1)

**Figure 2:**
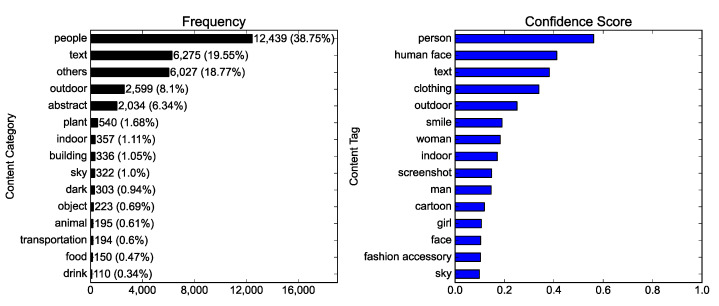
The overall content of the Instagram photos with a #NoMask hashtag: the frequency of photos by content category (**left**), and the content tags with the highest mean confidence scores (**right**).

The overall content of the Instagram photos with a #NoMask hashtag was examined to answer RQ1(a). Figure 2 reveals that people, especially human faces, were mainly represented; “people” was the category with the highest frequency, and “person” and “human face” were the content tags with the highest mean confidence scores. Other content tags such as “smile,” “face,” “clothing,” and “fashion accessory” also indicated that people and faces were the major objects represented in the photos. The surroundings of people in the photos were more outdoor than indoor: “outdoor” had a higher confidence score than “indoor.” Women’s faces appeared more in the photos than men’s: “woman” had a higher confidence score than “man”, and “girl” was also included in the content tags with the highest mean confidence scores. The “smile” tag indicated that the faces in the photos were generally smiling.

Additionally, Figure 2 also evinces that text was another major object represented in the photos: “text” was the category with the second-highest frequency and the content tag with the third-highest mean confidence score. Texts seem to appear in the photos through “screenshot” and “cartoon” which were included in the content tags with the highest mean confidence score.

The characteristics of the faces in the photos with a #NoMask hashtag were assessed to respond to RQ1(b). The number of photos containing at least one face totaled 13,050. Table 1 presents the mean facial features of those photos: the photos portrayed 1.441 faces on average and faces accounted for about 10% of the area of the photos (computed as 0.11 for closeup and 0.12 for face ratio). The faces in the photos represented young people aged around 31 years on average. The faces in the photos depicted females (0.855) more than males (1.441 − 0.855 = 0.586); this result is congruent with the women-related content tags (“woman” and “girl” combined) exhibiting higher confidence scores than the male-related one (“man”). Happiness was the strongest emotion expressed on faces, except for neutral; this result also corresponds to the findings described above on the “smile” content tag.

**Figure 3:**
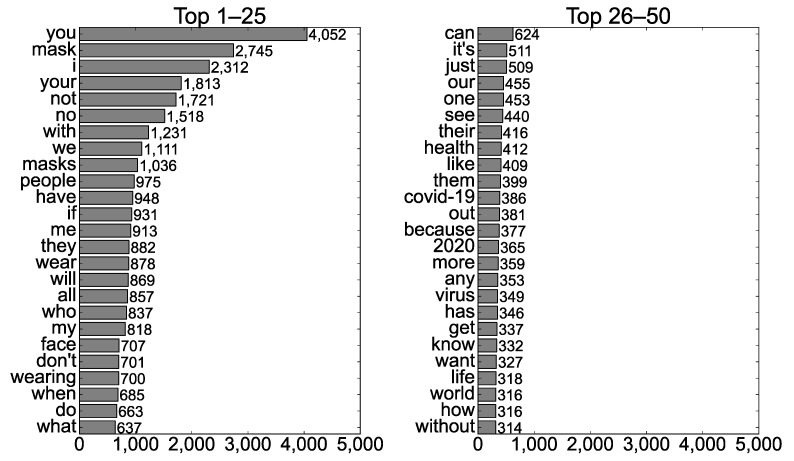
The 50 most frequent words presented in the Instagram photos with a #NoMask hashtag.

The words presented in the photos with a #NoMask hashtag were examined for RQ1(c), and Figure 3 displays the 50 most frequent words. Naturally, mask-related words such as “mask (s),” “face,” and “wear (ing)” appeared frequently. The context of a #NoMask hashtag was mentioned through words such as “COVID-19,” “virus,” and “2020.” #NoMask seemed to be utilized in terms of public health with words such as “health” and “life.” The high frequencies of negative words such as “not,” “no,” and “don’t” suggest that the negative aspects of wearing masks would primarily be asserted.

The most frequent word was “you,” indicating that the texts in the photos tended to directly address viewers. In addition, the frequent appearances of the words “if” and “because” suggested that the principal narrative technique was to assume a situation and to tender an explanation. These results demonstrate how key text messages were delivered in the photos with a #NoMask hashtag: writers assumed a situation (probably related to COVID-19) relating to masks in which readers could be involved or explained why viewers should or should not act in a certain manner.

### 3.2. Differences in Visual Representations between Clusters (RQ2)

**Figure 4:**
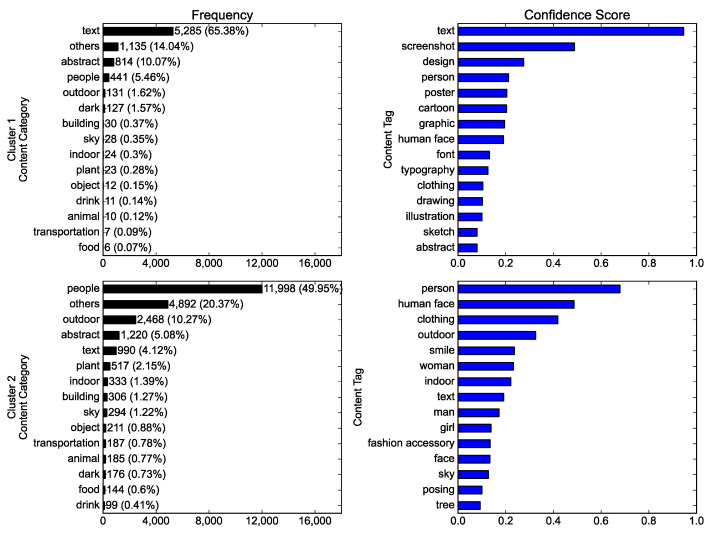
The comparison between clusters (upper vs. lower rows) of the Instagram photos with a #NoMask hashtag in terms of the content: the frequency of photos by content category (**left column**), and the content tags with the highest mean confidence scores (**right column**).

Two clusters were compared in terms of the content. Figure 4 shows the clear distinction between clusters: text-centered and people-centered. Cluster 1 yielded “text” as the content category with the highest frequency, and text-related tags such as “text,” “screenshot,” “design,” “font,” and “typography” exhibited high mean confidence scores. Contrastingly, photos assigned to the people category took the largest share of cluster 2, and people-related tags such as “person,” “human face,” “clothing,” “smile,” “woman,” “man,” and “girl” evinced high mean confidence scores.

The facial features visible in the photos designated to the two clusters were compared, and the results are presented in Table 2. Cluster 2 was people-centered and displayed more and bigger faces than cluster 1, with a larger number of faces and closeups, and greater face ratios. The faces in cluster 2 were older (larger age) and depicted more females (larger gender) than cluster 1. All emotions except anger and disgust were manifested more strongly in cluster 2; in particular, clear differences were evinced between clusters with respect to happiness and neutral expressions.

**Figure 5:**
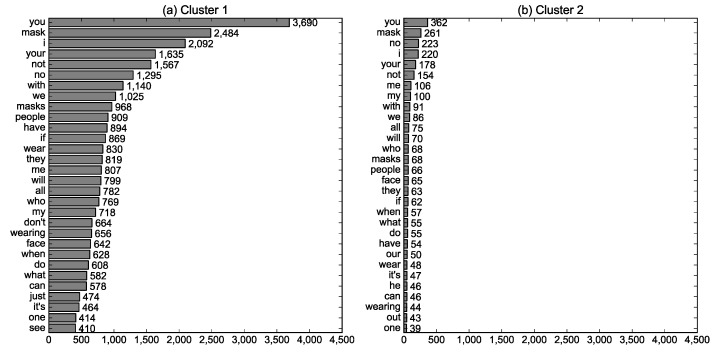
The 30 most frequent words in the Instagram photos with a #NoMask hashtag calculated by cluster.

The words presented in the photos classified into two clusters were compared, and Figure 5 presents the 30 most frequent words appearing in each cluster. Cluster 1 was text-centered and thus included more words: the mean number of words was calculated as 5.519 in cluster 1 and 0.398 in cluster 2 (t = 137.319, *p* = 0.000). Some words were peculiar to cluster 1 (“don’t,” “just,” and “see”) or cluster 2 (“our,” “he,” and “out”); however, obvious differences were not observed between clusters in presented words.

The pixel-level features of the two clusters were compared, and the results are displayed in Table 3. The photos in cluster 1 were more luminous; the RGB mean, value mean, and brightness were larger in cluster 1, and the contrast and RGB contrast were also greater than cluster 2. This outcome could be attributed to the bright backgrounds on which texts were presented in the text-centered photos in cluster 1. In contrast, the photos in cluster 2 were more colorful: their saturation mean and colorfulness were larger. Naturalness and sharpness were also larger in cluster 2. These findings could relate to the people, their activities, and the varied settings manifested in the photos of cluster 2, which portrayed more diversity in colors, more correspondence to the human perception of reality, and more detail.

Finally, the public reactions to the two clusters were compared. Cluster 2 demonstrated higher mean engagement (111.555) than cluster 1 (60.787), but the difference was insignificant (t = −1.819, *p* = 0.069). The mean comment sentiment was more positive for cluster 2 (0.198) than cluster 1 (0.073), and the difference was significant (t = −10.937, *p* < 0.001). These results indicate that the people-centered photos in cluster 2 received more positive comments than the text-centered photos in cluster 1, but the viewer engagement did not differ significantly for the two clusters.

## 4. Discussion

People, especially human faces, were most visually represented in the Instagram photos with a #NoMask hashtag. Most faces in the photos could be characterized as young women expressing happy or neutral emotions. The dominance of people and faces has already been reported in the literature on the content of Instagram photos from its early history [35] and for photos with diverse hashtags [57,58]. The outcomes of this study correspond to the reports of extant studies. However, the results concerning expressed emotions contradict the findings noted in the literature on mask aversion on SM. Some studies found negative emotions expressed in the rhetoric [19], linguistic characteristics [17], and themes [15], but another study identified positive sentiments in topics [16]. Notably, those previous studies analyzed text data, and the present study’s findings are yielded for the analysis of photo data: positive and neutral sentiments were mainly expressed in the Instagram photos related to mask aversion.

The Instagram photos with a #NoMask hashtag were grouped into two clusters: text-centered and people-centered. Their visual representations differed in content- and pixel-level characteristics. The photos in the text-centered cluster incorporated more words than the ones in the other cluster, and the background on which the texts were displayed increased the luminance and contrast of the photos. On the other hand, the photos placed in the people-centered cluster evinced more and larger human faces than the other cluster. The faces were older, expressed stronger emotions, and more of them portrayed females. The photos in this cluster were more colorful, natural, and sharp because of the appearance of people. These results can be meaningful because they manifested the visual representations in #NoMask Instagram photos in more detail. Two detected clusters showed differences in terms not only of the content but also the pixel-level characteristics, which convey signals and create meanings. In addition to the people-centered cluster that was larger in the number of photos and similar in characteristics to the overall dataset, there was a small but distinct text-centered cluster that played its role in visual representation. Additionally, text may bridge the gaps in visual representation by elucidating the meaning sought to be conveyed by the photos and by delivering more direct messages to the viewers. In this context, text has been reported in the extant literature to denote a major object in some types of Instagram photos [11,59]. This study’s results are aligned with such findings.

The texts presented in the photos seemed to deliver the key messages of the #NoMask hashtag in their distinctive way. The high frequencies of “you” and negative words suggest that the texts mentioned the negative aspects of wearing masks by directly addressing viewers. Additionally, the storytelling techniques used in the texts predominantly concerned assumption and explanation. These results are important because they demonstrated communication peculiarities of the texts presented in #NoMask Instagram photos that diverge distinctly from features observed in other hashtag movements. For example, a previous study identified the ways in which the antivaccination movement was presented in the texts of Instagram photos: they included remarks from professionals, presented key messages separately within the photos, and referenced sources to more information in the caption texts [11]. In addition, the results of the present study can be significant because most previous studies have analyzed caption texts [33] and texts presented in the photos have attracted limited attention from scholars.

In terms of the public’s responses, the photos placed in the people-centered cluster received more positive comments than the text-centered one. However, the two clusters did not exhibit significantly different public engagement. The existing literature reported that SM posts with human faces induced more engagement [60,61], but the present study cannot register a similar finding. The difference found in this study between clusters would be explained by the divergent modes of liking and commenting as reactions. Registering a “like” is an immediate reaction that needs little effort, but commenting involves writing which requires more mental and physical exertion than liking [43]. Thus, users could be more influenced by the characteristics of the photos when they post comments than when they press the like button. Consequentially, the happy emotion expressed in the faces would lead to more positive comment sentiments. Thus, the people-centered photos in cluster 2 naturally attracted more positive comments. In contrast, the negative words presented in the texts of photos in cluster 1 would trigger fewer positive comment sentiments, and this could explain why the photos in cluster 1, which were text-centered, attracted fewer positive comments.

## 5. Conclusions

SM functions such as hashtags and photo uploading can make user interactions faster and richer, but they can also facilitate the online spread of antisocial norms. Mask aversion is an antisocial norm disseminated on SM in the current COVID-19 pandemic. This study was grounded in SRT and it explored how mask aversion was visually represented in the Instagram photos with a #NoMask hashtag. In addition to the exploration of the entire dataset, visual representations were examined in subgroups identified via the k-means clustering algorithm. The results demonstrated the objects that were primarily depicted in the photos and how the representations differed according to the clusters.

The implications of this study concern its manifestation of how mask aversion is visually represented on Instagram. In so doing, the study contributes to a more comprehensive understanding of the dissemination of antisocial norms on SM. Theoretically, the current investigation expanded the scope of SRT to visual representation and to the hashtag movement on which scant SRT-based research has yet been conducted [33]. Methodologically, this study applied computational techniques to SR research. While extant studies have used computational methods to examine SRs in texts [62], SRs in visual data posted on SM have rarely been subjected to computational analysis. Additionally, this study attended to both the content and the pixel-level characteristics that function crucially in visual representations but have not attracted much attention in the existing literature. Practically, this study would be useful for detecting how antisocial norms are spreading online and devising appropriate intervention measures.

Admittedly, this study is limited by its analysis of only one exemplar of the online dissemination of antisocial norms. Diverse antisocial norms are spread online, and their dissemination could evince similarities with or differences from the outcomes of the current study. Prospective studies can reveal those characteristics. In particular, future researchers can explore how their visual representations differ or share similar features. Additionally, scholars can investigate how the SRs on a topic are expressed in similar or divergent ways verbally and visually and how such portrayals change over time.

## Figures and Tables

**Table 1:** The mean of facial features of the Instagram photos with a #NoMask hashtag.

Feature	Mean (SD)
Number of faces	1.441 (1.614)
Closeup	0.110 (0.132)
Face ratio	0.120 (0.134)
Age	30.997 (10.787)
Gender	0.855 (1.366)
Anger	0.014 (0.081)
Contempt	0.013 (0.061)
Disgust	0.002 (0.024)
Fear	0.002 (0.026)
Happiness	0.433 (0.436)
Sadness	0.019 (0.077)
Surprise	0.017 (0.087)
Neutral	0.499 (0.421)

**Table 2:** The mean comparison of facial features between clusters of the Instagram photos with a #NoMask hashtag.

Feature	Cluster 1	Cluster 2	t
Number of faces	0.320	0.675	−22.329 *
Closeup	0.011	0.056	−35.973 *
Face ratio	0.013	0.061	−36.738 *
Age	7.311	14.380	−33.475 *
Gender	0.153	0.413	−21.078 *
Anger	0.006	0.006	−0.229
Contempt	0.002	0.006	−8.861 *
Disgust	0.001	0.001	−1.013
Fear	0.001	0.001	2.079 *
Happiness	0.072	0.211	−31.517 *
Sadness	0.006	0.008	−3.888 *
Surprise	0.005	0.008	−3.236 *
Neutral	0.111	0.234	−26.594 *

* *p* < 0.05

**Table 3:** The mean comparison of pixel-level features between clusters of the Instagram photos with a #NoMask hashtag.

Feature	Cluster 1	Cluster 2	t
Red mean	150.756	125.352	41.639 *
Red variance	4953.540	4631.434	10.894 *
Green mean	145.916	116.136	50.536 *
Green variance	4939.018	4288.623	22.782 *
Blue mean	145.276	111.059	56.675 *
Blue variance	4771.863	4066.758	23.713 *
Saturation mean	60.708	81.059	−34.837 *
Saturation variance	3016.080	2936.486	2.759 *
Value mean	163.111	137.228	44.193 *
Value variance	4561.874	4435.141	4.540 *
Hue peaks	2.144	2.166	−1.577
Brightness	147.289	118.316	50.381 *
Colorfulness	35.170	40.213	−16.172 *
Naturalness	0.356	0.440	−15.394 *
Contrast	63.567	61.118	11.600 *
RGB contrast	115.070	111.426	10.112 *
Sharpness	7,4597.393	8,2896.601	−8.018 *

* *p* < 0.05.

## Data Availability

The data presented in this study are available on request from the corresponding author. The data are not publicly available due to privacy.
